# The Frequency of Methicillin-Resistant *Staphylococcus aureus* and *Coagulase* Gene Polymorphism in Egypt

**DOI:** 10.1155/2014/680983

**Published:** 2014-09-23

**Authors:** Hend M. Abdulghany, Rasha M. Khairy

**Affiliations:** Department of Microbiology and Immunology and Department of Biochemistry, Faculty of Medicine, Minia University, Minia 61511, Egypt

## Abstract

The current study aimed to use *Coagulase* gene polymorphism to identify methicillin-resistant *Staphylococcus aureus* (MRSA) subtypes isolated from nasal carriers in Minia governorate, Egypt, evaluate the efficiency of these methods in discriminating variable strains, and compare these subtypes with antibiotypes. A total of 400 specimens were collected from nasal carriers in Minia governorate, Egypt, between March 2012 and April 2013. Fifty-eight strains (14.5%) were isolated and identified by standard microbiological methods as MRSA. The identified isolates were tested by *Coagulase* gene RFLP typing. Out of 58 MRSA isolates 15 *coa* types were classified, and the amplification products showed multiple bands (1, 2, 3, 4, 5, and 8 bands). *Coagulase* gene PCR-RFLPs exhibited 10 patterns that ranged from 1 to 8 fragments with *AluI* digestion. Antimicrobial susceptibility testing with a panel of 8 antimicrobial agents showed 6 different antibiotypes. Antibiotype 1 was the most common phenotype with 82.7%. The results have demonstrated that many new variants of the *coa* gene are present in Minia, Egypt, different from those reported in the previous studies. So surveillance of MRSA should be continued.

## 1. Introduction

MRSA was identified as a hospital acquired pathogen in the 1960s. Infections with community-acquired MRSA (CA-MRSA) have emerged in the 1980s due to the spread of MRSA from hospitals to the community [1]. A highly pathogenic community-acquired organism different from those related to hospitals has emerged since the mid-1990s [2]. The increasing rate of CA-MRSA infections in many areas, coupled with the organism's unique pattern of virulence, clinical picture, and antimicrobial resistance, has important implications for treatment and infection control measures and acts as a serious challenge for the clinician [3]. The differentiation between CA-MRSA and hospital acquired MRSA (HA-MRSA) is becoming so difficult, since CA-MRSA could spread in hospitals [4].

Colonization is the first stage in the pathogenesis of MRSA infection. About 20% of people are considered as permanent carriers of* S. aureus*, mainly in the anterior nares and skin, and also axila, throat, intestine, perineum, and rectum may be colonized with* S. aureus*. About 30% of people are transient carriers, and 5 to 7% of them are colonized with MRSA [5].

Naturally* S. aureus* has shown variable genome structure that is associated with the variable strains in certain areas; those are responsible for the emerging of different epidemiologic profiles. Identification and subtyping of such strains is very important to apply suitable infection control programs tocontrol MRSAspread [6]. Both phenotyping and genotyping can be used to identify MRSA, and it is found that the cefoxitin disk diffusion test was 100% sensitive and specific for detection of MRSA [7]. Also various molecular typing techniques have been used, including pulsed-field gel electrophoresis (PFGE) and multilocus sequence typing (MLST), and these assays were considered as efficient techniques in MRSA typing but they are time consuming, expensive, and technically complex [8].

All strains of* S. aureus* produce coagulase enzyme, and its production can identify* S. aureus* infections [9]. DNA sequence analysis of the 3′-end of the* Coagulase* (*coa*) gene revealed heterogeneity in the 81 bp tandem repeats region that encoding repeated 27-amino-acid sequences in the C-terminal region. PCR amplification of this region showed DNA bands of different size and number that could be further differentiated by using restriction enzyme* AluI* [10]. Therefore, this assay is considered a simple and accurate subtyping method of* S. aureus* [11] and can be included in epidemiological studies and routine infection control programs [8].

The aim of this study was to use* coa* gene polymorphism to identify MRSA subtypes isolated from nasal carriers in Minia governorate, Upper Egypt, evaluate the efficiency of these typing methods in discriminating variable strains, and compare these subtypes with antibiotypes.

## 2. Methods

### 2.1. Bacterial Strains

A total of 400 specimens were collected from anterior nares of Egyptian carriers: two hundred from rural residents who are in contact with animals and 200 specimens from urban residents, between March 2012 and April 2013. One hundred fourteen (28.5%) isolates were identified as staphylococci, and fifty eight strains identified as* S. aureus* (32 from rural residents and 26 from urban residents) by standard microbiological methods including Gram stain, catalase, slide and tube coagulase test, DNase test, growth on mannitol salt agar, and complete hemolysis on blood agar [12].

### 2.2. Identification of MRSA

All* S. aureus* isolates were subjected to cefoxitin disc diffusion test to identify MRSA. Mueller-Hinton Agar plates were inoculated with 0.5 McFarland standard suspension of the isolates, and 30 *μ*g cefoxitin antibiotic disks were placed and incubated for 24 h at 37°C [13]. Zone diameters were measured and interpreted according to the guidelines of the Clinical Laboratory Standard Institute [14]. Fifty-eight (100%) strains were identified as MRSA and kept frozen at –20°C in brain heart infusion broth containing 15% glycerol for further identification.

### 2.3. Antimicrobial Susceptibility Testing

The sensitivity of MRSA isolates was examined by disk diffusion method based on CLSI guide direction, against antibiotics including vancomycin (30 *μ*g), ofloxacin (10 *μ*g), ampicillin (10 *μ*g), trimethoprim-sulfamethoxazole (25 *μ*g), amikacin (30 *μ*g), cefotaxime (30 *μ*g), amoxicillin/clavulanic acid (20/10 *μ*g), and clindamycin (2 *μ*g) (Bioanalyse).

### 2.4. DNA Extraction

DNA was extracted from the study samples and two isolates of methicillin-resistant coagulase-negative staphylococci (MRCoNS) that were included as negative control using genomic BYF DNA extraction Mini Kit (Intron Biotechnology, Korea); according to the manufacturer's instructions, DNA was stored at –20°C.

### 2.5. DNA Amplification

The 3′-end region of the* coa* gene was amplified by PCR using the following primer set [10]: forward: 5′CGAGACCAAGATTCAACAAG; the reverse: 5′AAAGAAAACCACTCACATCA 3′ (Biosearch Technologies). PCR was performed in a 50 *μ*L reaction mixture, containing 3 *μ*L of template DNA (approximately 300 ng/*μ*L) and 25 *μ*L of PCR master mix (DreamTaq TM Green Master Mix (2X), Fermentas). It is a ready-to-use solution containing DreamTaq TM DNA polymerase, optimized DreamTaq TM Green buffer (2X), 4 mM MgCl and dNTPs (dATP, dCTP, dGTP, and 2dTTP, 0.4 mM each), and 2 *μ*L (20 pmol) of each primer and 18 *μ*L of nuclease-free water. The reaction was carried out in a thermocycler (TECHINE TC 512) as follows: initial denaturation at 95°C for 5 min, followed by 30 cycles of denaturation at 95°C for 30 seconds, annealing at 55°C for 45 seconds, and extension at 72°C for 2 min, followed by a final extension at 72°C for 7 min.

### 2.6. Analysis of Restriction Fragment Length Polymorphism (RFLP)

Restriction analysis of the PCR products was performed with* AluI* enzyme (Thermo Scientific Fast Digest), according to the manufacturer's instructions as follows: 10 *μ*L of PCR products, 1 *μ*L of Fast Digest enzyme, 2 *μ*L of green buffer, and 17 *μ*L of nuclease-free water at 37°C for 15 minutes. The PCR products and* RFLP* fragments were identified by electrophoresis through 2% and 3% agarose gel using 100 bp ladder (Nippon Genetics Europe GmbH) and 50 bp ladder (QIAGEN Gelpilot), respectively.

### 2.7. Reproducibility Testing

PCR reproducibility was determined by testing 5 different isolates, for 5 consecutive days, and (*coa*-RFLP) reproducibility by analyzing 8 PCR products 3 times by* AluI* digestion.

### 2.8. Determination of Numerical Index of Discrimination

The discriminatory power of the typing method was determined according to the index described by Hunter and Gaston, 1988 [15]. The following formula is used:

(1) $$\begin{array}{c} D=1-\frac{1}{N\left(N-1\right)}\overset{s}{\underset{J=1}{\sum }}nj\left(nj-1\right), \end{array}$$

where *D* = discriminatory index, *s* = the total number of different types, *nj* = the number of each type isolates, and *N* = the total number of isolates.

## 3. Results

### 3.1. Antibiotypes

Six different antibiotypes (Ant 1–6) were identified in 58 different MRSA isolates using a panel of 8 antimicrobial agents (Table 1). Antibiotype 1 was the most common antibiotype, found in 82.7% of the isolates, and antibiotypes 2–6 were found in sporadic cases.

### 3.2. *Coagulase* Gene Typing

**Figure 1:**
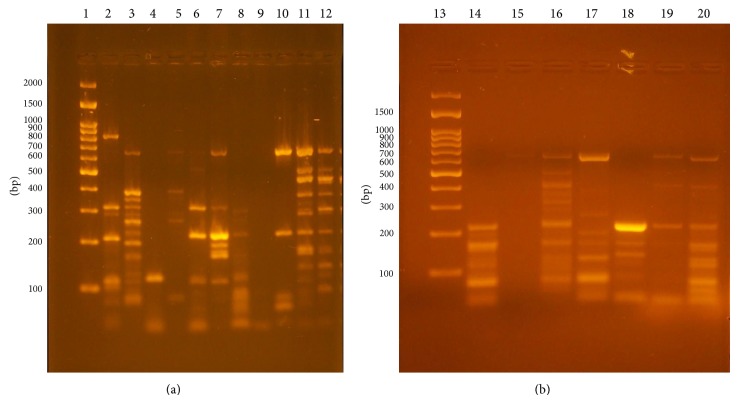
(a) Electrophoretic agarose gel images of* coa* gene PCR products of MRSA. Lane 1: 100 bp ladder, lane 2: C13: 5 bands (80, 110, 240, 320, and 810 bp), lane 3: C14: 8 bands (80, 110, 180, 240, 260, 320, 400, and 650 bp), lane 4: C1: 1 band (130 bp), lane 5: C8: 4 bands (80, 240, 400, and 650 bp), lane 6: C5: 3 bands (110, 240, and 320 bp), lane 7: C11: 5 bands (110, 180, 240, 320, and 650 bp), lane 8: C4: 2 bands (110 and 240 bp), lane 9 (negative control), lane 10: C6: 3 bands (80, 240, and 650 bp), and lanes 11 and 12: C15: 8 bands (110, 160, 180, 240, 320, 400, 480, and 650). (b) Lane 13: ladder, lane 14: C7: 3 bands (240, 160, and 80), lane 15: no product, lane 16: C12: 5 bands (650, 400, 240, 160, and 80), lane 17: C9: 4 bands (650, 200, 160, and 80), lane 18: C2: 2 bands (240 and 160), lane 19: C3: 2 bands (650 and 240), and lane 20: C10: 4 bands (650, 240, 160, and 80).

The* coa *gene was amplified in 58 different MRSA isolates from human nasal carriers and 2 MRCoNS strains. The size of the products ranged from approximately 80 to 810 bps, and these products showed 15 different types of band patterns as shown in Figures 1(a) and 1(b) and Table 2. Three MRSA isolates showed one band approximately 130 bp, eight isolates showed 2 bands that ranged approximately from 110 to 650 bps, eleven isolates had 3 bands approximately from 80 to 650 bps, fifteen isolates had 4 bands that ranged approximately from 80 to 650 bps, nine isolates had 5 bands that ranged approximately from 80 to 810 bps, and seven isolates showed 8 bands that ranged approximately from 80 to 650 bps. MRCoNS isolates and 4 isolates, identified as* Coagulase* positive by coagulase test, were found to be negative with PCR, so molecular detection of* S. aureus* strains is very important, and the typability by* coa* gene amplification was about 93% amongst the study isolates.

### 3.3. Restriction Fragment Patterns

**Figure 2:**
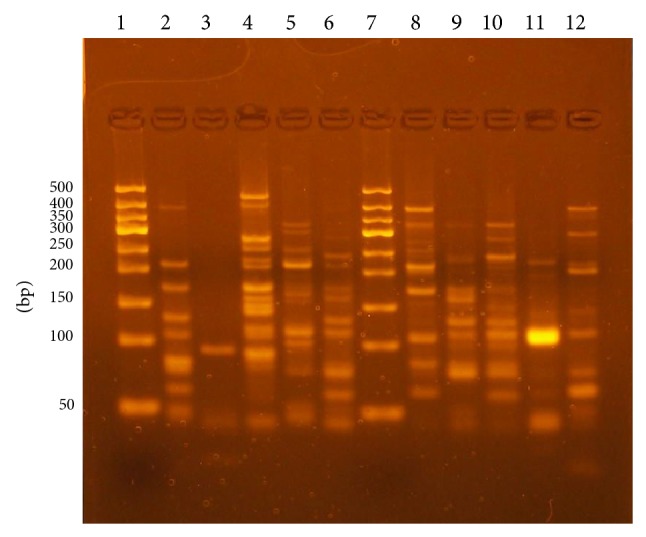
Electrophoretic agarose gel images of* AluI* restriction fragments of PCR products. Lane 1: 50 bp ladder, lane 2: A5 (7 fragments), lane 3: A1 (1 fragment), lane 4: A10 (6 fragments), lane 5: A8 (5 fragments), lane 6: A6 (7 fragments), lane 7: a ladder, lane 8: A9 (7 fragments), lane 9: A3 (4 fragments), lane 10: A7 (5 bands), lane 11: A2 (2 fragments), and lane 12: A4 (6 fragments).

The numbers of* AluI* RFLP patterns and genotype frequency are shown in Table 2. Ten different RFLP banding patterns were produced and typed as A1–A10. The isolates with one amplicon gave one pattern of digestion with 1 restriction fragment (80 bp), whereas those with 2 amplicons gave the same pattern of digestion with 2 fragments (240, 110 bp), and the isolates with 3 amplicons also gave the same pattern of digestion with 4 fragments (240, 160, 110, and 80 bp). The isolates with 4 amplicons gave 3 different patterns of digestion with 5 to 7 restriction fragments, and those with 5 amplicons gave 3 different patterns of digestion with 5 to 7 restriction fragments, whereas the isolates with 8 amplicons gave 2 different patterns of digestion with 6 and 7 restriction fragments (Figure 2 and Table 2). One isolate with 2 PCR amplicons did not show any restriction cut with* AluI*. This probably indicates the absence of* AluI* restriction site; therefore,* AluI* RFLP typability was 98% amongst these isolates.

The correlation between the genotypes, antibiotypes, and the source of the study isolates is summarized in Table 3 that shows variable* coa* types belong to the same antibiotype, C5 is present in 27% of MRSA isolates from urban residents, C8, C9, and C10 are predominant genotypes (46.9%) of MRSA isolates from rural residents, and C12 is isolated from rural and urban residents that suggest the source of the strain may be the contact with animals.

Reproducibility of the PCR products and* AluI* RFLP was detected with 100% of the repeatedly tested isolates. The amplicons were reproducible, although there was some variation in intensity of the bands.

The discriminatory index for the PCR-based typing and the* AluI* RFLP methods was about 0.93 and 0.90, respectively, which is a good result, so these methods are considered as efficient molecular typing methods.

## 4. Discussion

Typing of* S. aureus* by* coa *gene amplification has been considered to be technically simple, specific, and reproducible genotyping method [16] and effective in tracking the spread of infections so it can shorten or prevent an epidemic and determine the source of infection that decreases the morbidity and mortality rates [8].

In the current study, MRSA strains were 14.5% carriage and 100% of* S. aureus* isolates that disagreed with most of the previous studies (Brannon et al., 2009) [5] that reported that 7.5% MRSA carriage and 25% of* S. aureus* strains were MRSA. Tiwari et al., 2008 [17], also reported different results that 64% of* S. aureus* strains in his study were identified as MRSA.

This study has reported 4 different antibiotypes and showed 100% sensitivity to vancomycin and variable response to different antibiotics that agreed with most of the previous studies, [5, 18].

Previously,* coa *gene amplification was reported to produce single-banded PCR products in* S. aureus* strains isolated from human and animal samples, and double-banded products also have been detected [10, 19, 20], but this was a rare finding. After that, double and triple bands from human samples were reported [17]. Triple bands were also reported in Egypt [21] from human and animal samples.

The current study has reported a completely different finding: multiple bands amplification products (1, 2, 3, 4, 5, and eight bands) were detected. According to these findings the studied strains were classified as 15* coa *PCR types, and this agreed partly with E. R. da Silva and N. da Silva [20], who reported 27 types among 64 isolates, and disagreed with the most of other researchers: Janwithayanuchit et al. [18], who determined 4 different patterns of* coa* gene in 129* MRSA* isolates, Himabindu et al. [8], who reported 3 classes among 85 isolates, Demir et al. [22], who reported 4 patterns in 120 isolates, and Talebi-Satlou et al. [23], who reported 4 products in 26 isolates.

The variability in size and number of* coa* bands detected in this study may be due to the presence of different allelic forms of* coa *gene in MRSA, allowing one strain to produce multiple amplicons [10].

This study has reported 10* AluI* RFLP patterns amongst 54 isolates, and this was compatible with that reported in Japan, 6 different RFLP banding patterns with* AluI*, in 35 MRSA isolates [24], and that reported in India, 31 distinct patterns in 85 isolates that indicate MRSA has considerable heterogeneity in the studied group [8]. Our finding disagreed with Demir et al. [22] who reported 7 patterns in 120 isolates.* AluI* PCR-RFLP fragments in our study varied from one to seven bands, and these results differ from the previous studies, in which* AluI* PCR-RFLP fragments varied from one to five bands using the same primer sequences [8, 25]. So the current study has reported novel and unique results. One isolate did not show any restriction cut that agreed with the study that has reported no cut in four isolates [8].

We have reported about 93% and 98% typability of* coa* gene amplification and* AluI* PCR-RFLP amongst the study isolates, respectively. This agreed to some extent with studies that reported 97% typability [20].

Our study has reported that the discriminatory index of both PCR-based and the* Alu*I RFLP typing methods was about 0.95 and 0.90, respectively. Other studies have reported discriminatory index of 0.92 for PCR amplification, 0.99 for* coa*-RFLP analysis [18], 0.55 based on PCR product sizes, and 0.96 for AluI typing method [8].

## 5. Conclusion

This study has shown that MRSA strains colonize the carriers in the studied area harboring different and novel* coa *genotypes. We would recommend PCR-RFLP typing method as an efficient practical method in infection control programs because of its high discriminatory power and typability that can distinguish between the epidemic and sporadic strains during outbreaks. Further studies should use a large number of isolates and cover more wide areas.

## Figures and Tables

**Table 1:** Susceptibility of antimicrobials.

Antibiotype	Susceptibility of antimicrobials								Number (%)	Source
	VAN	DA	AMK	AMC	OFX	AMP	CX	SXT		
Ant 1	S	S	S	R	S	R	S	S	48 (82.7%)	Urban/rural
Ant 2	S	S	S	R	S	R	S	R	2 (3.4%)	Urban
Ant 3	S	S	R	R	S	R	S	R	2 (3.4%)	Urban
Ant 4	S	S	S	S	S	R	S	S	1 (1.7%)	Rural
Ant 5	S	S	S	R	R	R	S	S	2 (3.4%)	Rural
Ant 6	S	S	R	R	R	R	S	R	3 (5.2%)	Rural

Abbreviations for susceptibility: R: resistant; S: susceptible.

Abbreviations for antimicrobial agents: SXT: trimethoprim-sulfamethoxazole, AMK: amikacin, DA: clindamycin, AMC: amoxicillin/clavulanic, OFX: ofloxacin, VAN: vancomycin, AMP: ampicillin, and CX: cefotaxime.

**Table 2:** Frequency of coagulase genotypes in the MRSA isolates.

Type	PCR products (bps)	Number	Pattern	RFLP fragments	Frequency
C1	130	3	A1	80	5.5
C2	240 and 160	4	A2	240, 110	7.4
C3	650 and 240	3	A2	240 and 110	5.5
C4	240 and 110	1	—	No	1.8

C5	320, 240, and 110	7	A3	240, 160, 110, and 80	12.9
C6	650, 240, and 80	2	A3	24, 160, 110, and 80	3.7
C7	240, 160, and 80	3	A3	240, 160, 110, and 80	5.5

C8	650, 400, 240, and 80	5	A4	400, 320, 240, 110, 80, and 60	9.3
C9	650, 200, 160, and 80	5	A5	400, 240, 160, 130, 110, 80, and 60	1.8
C10	650, 240, 160, and 80	5	A6	240, 160, 110, 80, and 60	16.6

C11	650, 320, 240, 180, and 110	2	A6	240, 160, 110, 80, and 60	3.7
C12	650, 400, 240, 160, and 80	5	A7	320, 280, 240, 160, 110, 80, and 60	9.3
C13	810, 320, 240, 110, and 80	2	A8	320, 240, 160, 110, and 80	3.7

C14	650, 400, 320, 240, 200, 160, 110, and 80	3	A9	400, 320, 240, 160, 120, 80, and 60	1.8
C15	650, 480, 400, 320, 240, 180, 160, and 80	4	A10	480, 280, 240, 160, 110, and 80	11.1

**Table 3:** Correlations between antibiotypes, PCR-based types, and source of strains.

Antibiotypes (*n*)	Coagulase gene type (*n*)	PCR-RFLP pattern	Source
1 (4)	— (4)	— (4)	Rural
1 (3)	C1 (3)	A1 (3)	Urban
1 (4)	C2 (4)	A2 (4)	Urban
1 (3)	C3 (3)	A2 (3)	Urban
1 (7)	C5 (7)	A3 (7)	Urban
1 (2)	C6 (2)	A3 (2)	Rural
1 (3)	C7 (3)	A3 (3)	Rural
1 (5)	C8 (5)	A4 (5)	Rural
1 (5)	C9 (5)	A5 (5)	Rural
1 (5)	C10 (5)	A6 (5)	Rural
1 (2)	C11 (2)	A6 (2)	Urban
1 (2)	C12 (2)	A7 (2)	Rural
1 (2)	C12 (2)	A7 (2)	Urban
1 (1)	C14 (1)	A9 (1)	Urban
2 (2)	C15 (2)	A10 (2)	Urban
3 (2)	C15 (2)	A10 (2)	Urban
4 (1)	C4 (1)	— (1)	Rural
5 (2)	C13 (2)	A8 (2)	Rural
6 (2)	C14 (2)	A9 (2)	Rural
6 (1)	C12 (1)	A7 (1)	Rural
